# Phase Transformation and Morphology Evolution of Ti_50_Cu_25_Ni_20_Sn_5_ during Mechanical Milling

**DOI:** 10.3390/ma11091769

**Published:** 2018-09-18

**Authors:** Dora Janovszky, Ferenc Kristaly, Tamas Miko, Adam Racz, Maria Sveda, Anna Sycheva, Tomasz Koziel

**Affiliations:** 1 MTA-ME Materials Science Research Group, H-3515 Miskolc, Hungary; femmaria@uni-miskolc.hu (M.S.); femanna@uni-miskolc.hu (A.S.); 2 Institute of Mineralogy and Geology, University of Miskolc, H-3515 Miskolc, Hungary; kristalyf@gmail.com; 3 Institute of Physical Metallurgy, Metalforming and Nanotechnology, University of Miskolc, H-3515 Miskolc, Hungary; femmiko@uni-miskolc.hu; 4 Institute of Raw Material Preparation and Environmental Processing, University of Miskolc, H-3515 Miskolc, Hungary; ejtracz@uni-miskolc.hu; 5 Faculty of Metals Engineering and Industrial Computer Science, AGH University of Science and Technology, 30-059 Krakow, Poland; tkoziel@agh.edu.pl

**Keywords:** amorphous–nanocrystalline composites, Ti-based amorphous alloys, ball milling, powder metallurgy, microstructure, mechanical properties

## Abstract

Nanocrystalline/amorphous powder was produced by ball milling of Ti_50_Cu_25_Ni_20_Sn_5_ (at.%) master alloy. Both laser diffraction particle size analyzer and scanning electron microscope (SEM) were used to monitor the changes in the particle size as well as in the shape of particles as a function of milling time. During ball milling, the average particle size decreased with milling time from >320 µm to ~38 µm after 180 min of milling. The deformation-induced hardening and phase transformation caused the hardness value to increase from 506 to 779 HV. X-ray diffraction (XRD) analysis was used to observe the changes in the phases/amorphous content as a function of milling time. The amount of amorphous fraction increased continuously until 120 min milling (36 wt % amorphous content). The interval of crystallite size was between 1 and 10 nm after 180 min of milling with 25 wt % amorphous fractions. Cubic Cu(Ni,Cu)Ti_2_ structure was transformed into the orthorhombic structure owing to the shear/stress, dislocations, and Cu substitution during the milling process.

## 1. Introduction

Among the metallic elements of the periodic system, lightweight Ti occupies a special position due to its excellent high-temperature oxidation resistance and biocompatibility. In addition, it also has favorable mechanical properties, which—as in case of conventional crystalline materials—depend on the grain size (Hall–Petch relationship), with Ti-based amorphous alloys possessing ultrahigh strength and hardness [1,2,3]. Unfortunately, the bulk amorphous alloys still do not display sufficient ductility for industrial applications. In most application fields, users expect materials to have both high tensile strength and high tensile ductility. However, materials usually have high strengths but low ductility, and it is very rare for both parameters to be high at the same time. A good solution in overcoming this strength–ductility paradox is the fabrication and employment of a bimodal alloy: nanosized particles dispersed in amorphous matrix. The extreme enhancement in strength has been realized with ductility in nanocrystalline/amorphous alloys [4,5,6,7].

Along with Ti-based, fully amorphous alloys, the amorphous alloys with nano- or microsized particulate composites have attracted much attention, due to their high specific strength (tensile strength higher than 1800 MPa) with lower density (4.5 g/cm^3^) [8,9]. Amorphous and nanostructured materials have attracted extensive interest because they possess unique properties that can rarely be found in microcrystalline materials [1,2,3,4,5].

Amorphous metals with nanostructured particles can be produced by different techniques. Rapid solidification is a useful method to achieve an amorphous + nanocrystalline microstructure [10]. Amorphous–nanocrystalline structure can also be achieved by controlled crystallization of fully amorphous structure [11,12]. However, both the abovementioned methods are expensive. As for solid-state techniques, mechanical alloying (MA) is one of the most useful techniques for the production of nanocrystalline alloys and amorphous systems [13,14,15]. However, the MA process is a time-consuming process that usually takes more than 10–20 h. As a result of the long grinding time, Fe contamination can be a serious problem. One of the alternative procedures to produce amorphous and nanostructured alloy is mechanical milling (MM) [16,17]. Despite this process taking shorter time than MA, there have been limited studies of the amorphous and nanocrystalline compositions produced by MM.

Considering all aspects mentioned above, this work is focused on the synthesis of a Ti-based amorphous and nanocrystalline composite. Ti_50_Cu_25_Ni_20_Sn_5_ was chosen as the Ti-based bulk amorphous alloy composition for our study [18].

The other aim of this work was to study the process of phase transformations during high energy milling. The transformation of crystalline phases is usually not investigated in detail, with studies mainly focusing on the level of amorphous phase generation and disappearance of crystalline components [19,20]. Although the development of new crystalline phases by MA is frequently examined (by XRD or High-resolution transmission electron microscopy (HRTEM)), the transformations by MM have mostly been disregarded. Powder XRD is a suitable method to analyze larger volumes of MM samples, giving an average result of all particle (crystallite) characteristics. Using this method and applying Rietveld refinement, it is therefore possible to quantify the developed amorphous content as well as the type and amount of amorphized crystalline phases [21,22].

## 2. Materials and Methods

Master alloy ingot with the composition Ti_50_Cu_25_Ni_20_Sn_5_ (at.%) was prepared by arc melting of pure metal mixtures (min. 99.9 wt %) with a Ti-getter under purified argon atmosphere. It was impossible to crush the as-cast ingots into fine chunks (or coarse powder) for further ball milling, even using a heavy sledgehammer. Therefore, the ingots were machined by a turning mill into chips. We used a tungsten carbide tool without any lubricant in order to decrease the amount of contamination of the chips.

The machined chips were fractioned to a particle size below 320 μm for planetary ball milling. Amorphous/nanostructured powders were obtained in a Pulverisette 5 high-energy planetary ball-mill (Fritsch Gmbh, Weimar, Germany) in argon atmosphere using a 250 mL stainless steel grinding bowl and hardened steel balls of different diameters, which were used together. The diameters of the hardened steel balls were 20 mm, 15 mm, 10 mm, 5 mm, and 2.5 mm. Ball-to-powder weight ratio and rotational speed were set at 80:1 and 200 rpm, respectively. The angular velocity of the grinding bowl and the supporting disc were approximately 231 and 200 rpm, respectively. The initial weight of the powder was 6.4 g. To study the effect of milling time, samples were taken between 30 and 180 min by interrupting the milling cycle. A sample of 0.5 g was taken every 30 min to check the effect of the milling time on the particle size, shape, and amorphous content.

The micrographs of the powders were acquired by a Hitachi S-4800 scanning electron microscope (SEM, Hitachi, Tokio, Japan) equipped with a BRUKER AXS type energy-dispersive X-ray spectrometer (EDS, Bruker Gmbh., Berlin, Germany). Backscattered electron micrographs (BSE) were recorded in order to get information about the microstructure of the samples. The particle size distribution of the ground material was determined by a Horiba LA-950 V2 type laser diffraction particle size analyzer (LPSA, Horiba Ltd., Kyoto, Japan) in distilled water. During the measurement process, 1-min ultrasonic treatment and 1 mL of 50 g/L sodium pyrophosphate dispersant were applied to achieve the appropriate dispersity state.

Thermal analysis was performed in a Netzsch 204 DSC (Netzsch Ltd., Selb, Germany) at a heating rate of 0.66 K/s under flow of purified argon.

The samples were examined by a Bruker D8 Advance diffractometer (XRD, Bruker Gmbh., Berlin, Germany) using Cu Kα radiation (40 kV, 40 mA) in parallel beam geometry obtained with Göbel mirror, equipped with Vantec-1 position sensitive detector (1° window opening) and measured in the 2–100° (2θ) angular range, with a 0.007° (2θ)/29 s speed. The specimen was rotated in the sample plane during the measurement to obtain data from the whole surface and to reduce in-plane preferred orientation effects. The amorphous fraction was determined by XRD analysis using peak area determination in TOPAS4 (amorphous hump method). The quantitative results were obtained by combined use of Rietveld refinement and peak area calculation (Pawley fit).

The microhardness measurements were performed by Instron Tukon 2100B equipment (Instron, Grove, OK, USA), applying a load of 10 g for 15 s for as-milled powders. The powder samples were embedded in acrylic resin and polished to produce a surface suitable for microindenting. Only the symmetric indenting marks were used to calculate HV_001_ values in order to avoid misleading results from indenting of aggregates.

## 3. Experimental Results and Discussion

**Figure 1:**
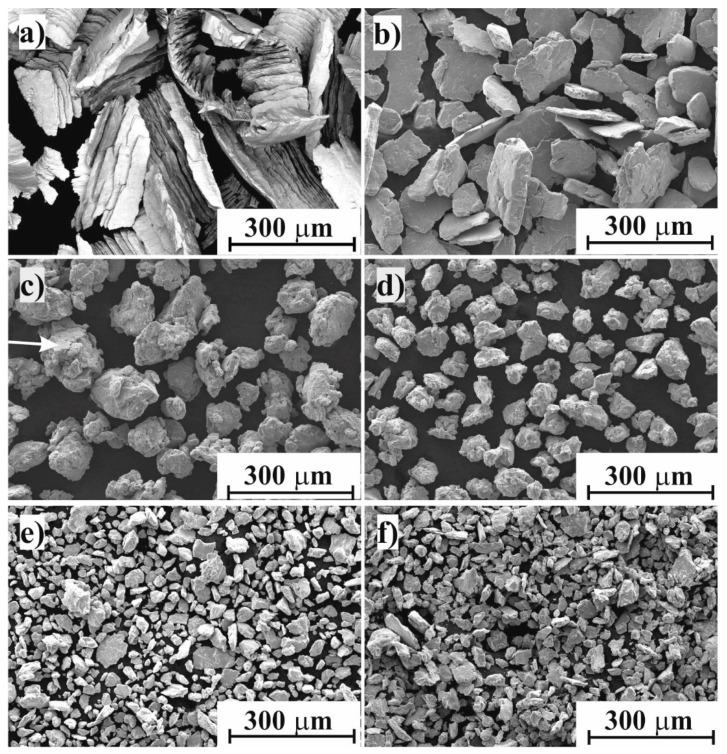
Scanning electron microscopy (SEM) images for the Ti_50_Cu_25_Ni_20_Sn_5_ alloy milled for different periods: (**a**) the as-received chips and after (**b**) 30 min, (**c**) 60 min, (**d**) 120 min, (**e**) 150 min, and (**f**) 180 min of milling.

The morphology of as-received machined chips is shown in Figure 1a. A very strong anisotropy was developed; the shape of the chips was short and discontinuous with narrow, straight primary shear zones, resulting in arrays of thin lamellar subparticles of 30 × 1 µm. The average thickness and length of the chips were ~30 µm and ~500 µm, respectively. The hardness of the chips was HV_0.01_ 506. Figure 1b shows the SEM micrograph of the chips after 30 min of ball milling. It can be seen that the subparticles had become separated into the flattened, thin disk-shaped particles due to shearing effect of the balls. The “saw tooth”-like shape observed on the primary chips particles disappeared, indicating that only the detachment of thin lamellar subparticles occurred without further shearing or deformation. The shape of particles was still angular, with minimal rounding of the corners and edges.

**Figure 2:**
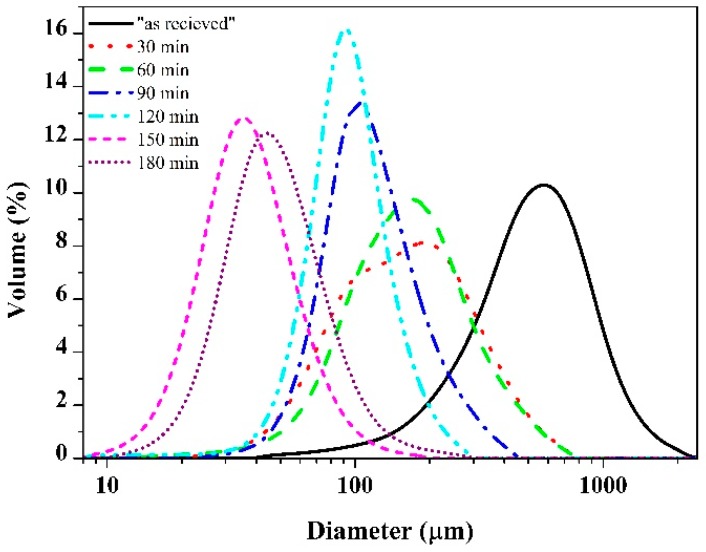
Particle size distribution of the Ti_50_Cu_25_(Ni_80_Sn_20_)_25_ powders after different milling times.

**Figure 3:**
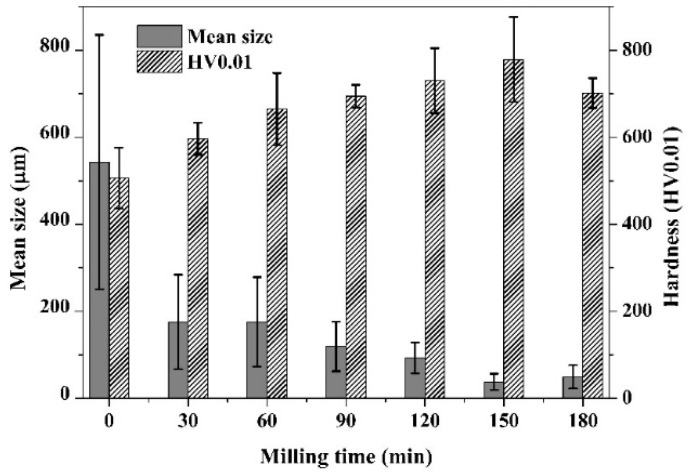
Effect of milling time on the average particle size and hardness.

Therefore, the median particle size (163 µm) of the sample after 30 min milling had decreased significantly compared to the initial particle size (543 µm) (Figure 2), but the thickness of particles was comparable to the subparticles of the as-received chips (Figure 1b). After 60 min of ball milling, the shape of particles changed to a spherical one, with significant rounding and deformation of lamellar particles. Besides rounding and fragmentation, aggregation of particles was also observed. Figure 1c shows the aggregates of small particles, still preserving partial lamellar shapes on the surface of aggregates (Figure 1c marked by an arrow). The reason for this coupled fragmentation–aggregation was that the powder particles were trapped between the colliding balls due to significant degree of plastic deformation [23]. The result of this complex physicochemical process was the attachment and binding of particles, known as cold welding, and it was observed for powder/powder and powder/ball surfaces as well. The action of cold welding was also observed on LPSA (Figure 2); the slightly bimodal distribution at 30 min became uniform at the median of the 30 min milled particle size. One possible explanation of the cold welding was the formation of active surfaces due to collisions, such as mechanical activation and plastic deformation. Additionally, the lack of oxygen and surfactant during the collisions promoted the cold welding [24]. Despite the fact that the shape of the particles had changed drastically, the average particle size distribution had not changed significantly (Figure 3). It is noteworthy that a small fraction of larger lamellar particles was still preserved.

After 90 and 120 min of milling, the mean particle size was continuously decreasing, owing to the dominance of fracturing over the cold welding that was still taking place. The majority of particles of 120 min milled powder showed the same sphere-like nature as at 60 min (Figure 1d) but of significantly smaller size. After 150 min of ball milling, the shape of particles changed again to the flattened, thin, disk-shaped ones due to the shearing effect of balls that destroyed the aggregates (Figure 1e). The small, thin lamellar particles were liberated from the spherical aggregates. The disaggregation was also observed by LPSA with a severe decrease in median particle size (Figure 2). A small but insignificant coarsening was produced up to 180 min. The hardness of particles increased continuously up 150 min of milling, with a HV_0.01_ 779 (Figure 3) peak value. The 1.5× increase in hardness was produced by the development of high dislocation density [17].

**Figure 4:**
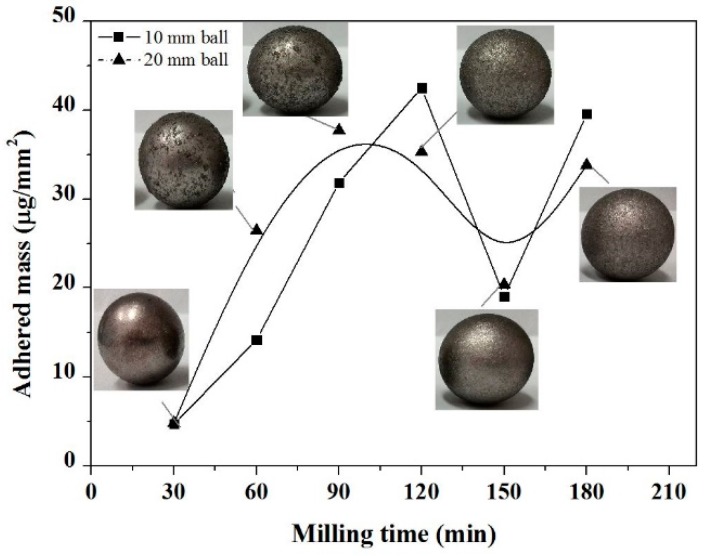
Effect of milling time on the value of adhered mass on the surface of milling balls.

The effect of cold welding by ball milling was also observed by measuring the mass of adhered powder on the balls surface [25]. In the early stage of the milling, the particles suffered plastic deformation due to repeating impact force of the balls. New particle surfaces were created that enabled the adhesion between the impacted particles, and a coating was formed on the balls. The maximally adhered mass value on the balls surface (Figure 4) was measured after 60 min of milling, which confirmed the presence of adhesion. In the period from 60 to 150 min of milling, the adhered mass value decreased. This phenomenon results from the increased hardness of powder particles as well as the disaggregation. The thin film of powder on the balls became brittle and peeled off. From a milling time of 150 min, the adhered powder mass increased again owing to the decrease in hardness.

**Figure 5:**
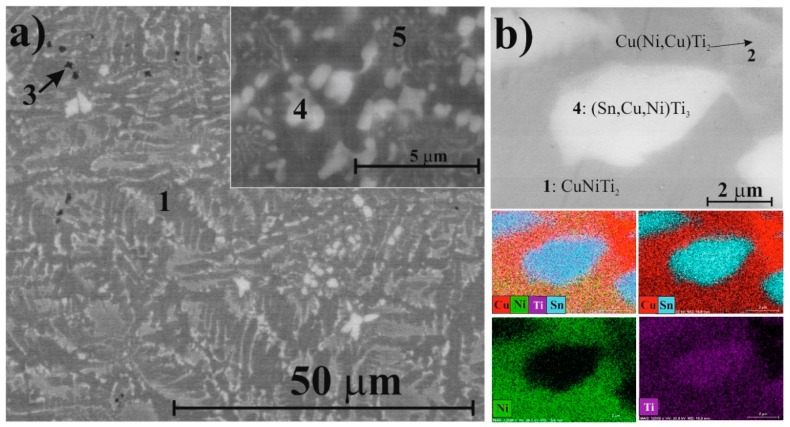
Backscattered SEM images (**a**) and characteristic compositional XEDS-SEM mapping of the same area (**b**) for the Ti_50_Cu_25_Ni_20_Sn_5_ master alloy.

Based on the XRD patterns and SEM analysis, five phases were identified in the master alloy of Ti_50_Cu_25_Ni_20_Sn_5_ composition. CuNiTi_2_ dendrites (SEM) with cubic structure (XRD) solidified first from the cooling melt (Figure 5a,b, marked as 1). According to XRD, the CuNiTi_2_ phase was present with predominantly cubic and orthorhombic crystal structures. Integrating EDS and XRD–Rietveld results, we observed that in the orthorhombic type, some Ni atoms were partially replaced by Cu without modifying the crystal structure (Table 1). Thus, hereinafter, this orthorhombic phase was noted as Cu(Ni,Cu)Ti_2_ (Figure 5b, marked as 2); the chemical composition calculated from the Rietveld refinement was Cu(Ni_0.7_Cu_0.3_)Ti_2_. A dark phase (Figure 5a, marked as 3) observed in the BSE image (according to EDS, 92–95 at. % on average was Ti, the rest was Cu) corresponding to the hexagonal α-Ti phase was also identified in the master alloy. Because some Cu was dissolved in Ti, this phase was hereinafter referred to as α-Ti(Cu) in XRD results. The composition of this phase calculated from the Rietveld refinement was Ti_0.8_Cu_0.2_ (Table 1). Two Sn-containing phases were also observed by SEM, EDS, and identified in the XRD patterns. According to the unit cell data, one phase (Figure 5a,b, marked as 4) was the modification of the hexagonal SnTi_3_ phase. Theoretical unit cell dimensions of the SnTi_3_ phase were a = 0.5916 nm, c = 0.4764 nm [26]. However, the Rietveld refinement showed a = 0.5894 nm, c = 0.4759 nm, indicating that the crystal structure was distorted. This phenomenon occurred when Sn was replaced by Cu and Ni atoms, which have smaller atomic radii (r_Cu_ = 0.1278 nm, r_Ni_ = 0.1246 nm) compared to Sn (r_Sn_ = 0.1620 nm). The composition of this phase calculated from the Rietveld-refinement was (Sn_0.4_Cu_0.35_Ni_0.25_)Ti_2.91_, which was in partial agreement with the EDS (Sn_18_Cu_6_Ni_4_Ti_72_) results. This phase was hereinafter referred to as (Sn,Cu,Ni)Ti_3_ phase in the XRD results. According to XRD, the other Sn-bearing component identified by EDS (Figure 5a, marked as 5) was the modification of the tetragonal CuTi_3_ phase. The composition of this phase calculated from the Rietveld-refinement was (Cu_0.7_Sn_0.3_)Ti_3_, where Sn atoms replaced Cu atoms; this phase was hereinafter referred to as (Cu,Sn)Ti_3_ (Table 1). The EDS measurement could not be performed as the size of this phase was smaller than the excitation volume and because it was solidified in eutectic with the CuNiTi_2_ phases. According to the Rietveld refinement, the total fraction of crystalline phases was 97 wt % (3 wt % amorphous) in the starting material, with all the phases being below 20 nm in crystallite size. The cubic CuNiTi_2_ dendrites and (Sn,Cu,Ni)Ti_3_ had the largest crystallite sizes, while (Cu,Sn)Ti_3_ had the smallest crystallite size (Table 1).

**Figure 6:**
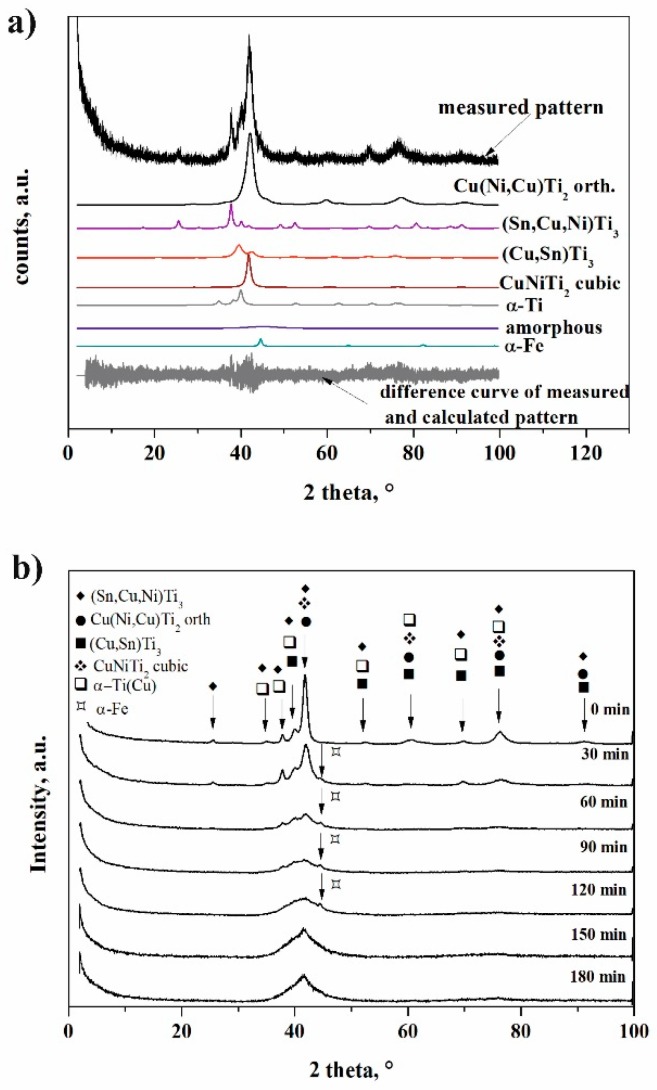
X-ray diffraction patterns of powder (**a**) after 30 min of milling and (**b**) powders with different milling time.

**Figure 7:**
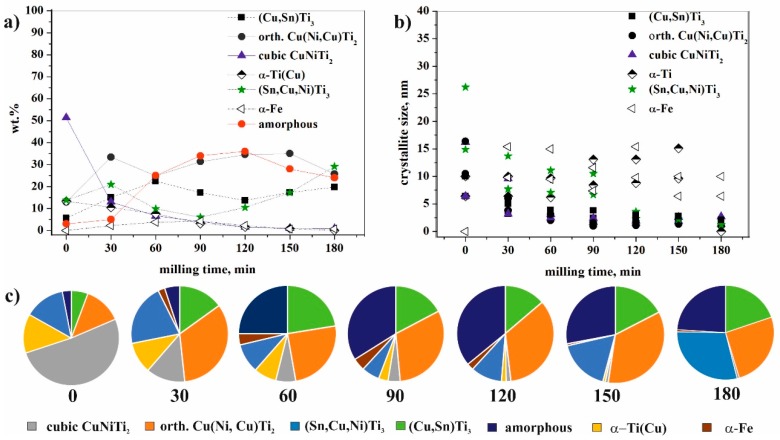
Fraction and featuring of (**a**,**c**) crystalline and amorphous phases and (**b**) crystallite size in the course of the milling process of Ti_50_Cu_25_Ni_20_Sn_5_ alloy determined by XRD measurement.

The phase evolution of powders that was occurring by ball milling was followed by the Rietveld refinement (Figure 6). Example of the phase determination for the Ti_50_Cu_25_Ni_20_Sn_5_ specimen is shown in Figure 6b. The most important process was the evolution of amorphous phase(s), based upon which we delimited three stages of reactions:

1. 0 to 30 min—activation period, which was dominated by particle rearrangement (Figure 1b) and crystalline transformations, with minor amorphization (Figure 7) and without cold welding.
2. 30 to 120 min—amorphization period, during which the milled powder was mainly composed of nanocrystalline phases. Accumulation of various crystal defects played a dominating role, and an increase in the storage energy of milled sample by crystal defects was evidenced by strong cold welding. The amorphization was enhanced through the destruction of nanocrystalline phases.
3. Over 120 min—recrystallization period, when reduction of the storage energy of milled sample by atomic migration/recrystallization dominated and then the amorphous phase transformed back into nanocrystalline phase.

One significant index of fitting results is R-Bragg, a goodness of fit index for single phase calculations that is a dimensionless number; the best fit is given by the value of 1. Our results had good R-Bragg values—well within common values for the Rietveld refinement—except for 120 and 150 min milling, which coincided with the recrystallization period. This fact suggests that nanocrystalline phases might be higher in number but similar in structure to those observed by XRD, e.g., phases developed in high dislocation density zones and grain boundaries. However, identification and quantification of these phases is beyond the field of XRD and requires more detailed investigations, which is out of the scope of this paper.

The changes in the amount of the phases, as a function of the milling time, offered new insights (Table 1) into the phenomenon (Figure 7a,c). A very significant change occurred with the cubic CuNiTi_2_ phase, with its amount exponentially decreasing during milling. After 180 min of milling, the fraction was below 1 wt % because this structure transformed into orthorhombic Cu(Ni,Cu)Ti_2_ owing to the stress and Cu substitution in the course of the milling process. The same transformation of BCC to orthorhombic structures has been previously published for a Ti–Al–Nb system [27]. The amount of the orthorhombic Cu(Ni,Cu)Ti_2_ phase increased within 120 min of milling. The length of “a” unit cell of this orthorhombic phase continuously decreased, while the “b” and “c” unit cell lengths were constant from 30 min of milling (Table 1). The decrease in the unit cell for “a” edge indicated more pronounced effect of Cu replacing Ni, which was also confirmed by the decrease in unit cell volume. The crystallite size of the orthorhombic Cu(Ni,Cu)Ti_2_ phase decreased exponentially from 10.5–16.4 nm to ~1.0 nm (Figure 7b) at which size the material should be considered, in our view, in a state between nanocrystalline and amorphous, “short range ordered phases” [28]. The total amount of cubic and orthorhombic structures was not constant—it decreased from 64 wt % to 31 wt % within 60 min of milling, while the fraction of amorphous phase(s) increased from 3 wt % to 25 wt % (Figure 7a,c). This could be explained if the Ti, Cu, and Ni atoms of these two structures had at least partly transformed into “X-ray amorphous structure” (hereinafter referred to as amorphous structure), simultaneously for both crystalline phases. In the period from 90 and 150 min of milling, the total amount of these structures was almost constant. However, its quantity significantly decreased after 150 min of milling (Table 1).

The amount of the hexagonal α-Ti(Cu) phase continuously decreased, and this phase disappeared after 180 min of milling.

**Figure 8:**
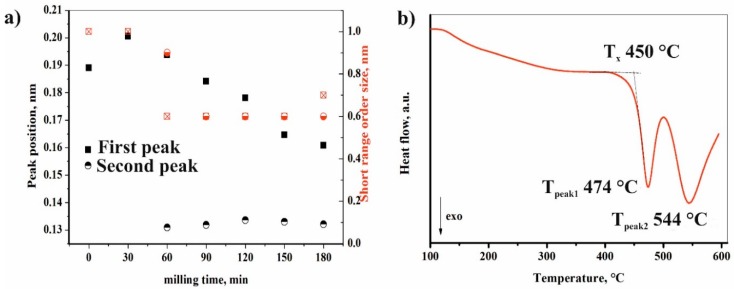
Effect of milling time on features of amorphous content (**a**) and constant heating rate (40 °C/min) DSC trace; (**b**) obtained for Ti_50_Cu_25_Ni_20_Sn_5_ alloy after 180 min of milling.

The change in the amount of both the Sn-containing phases was very noticeable (Table 1). The amount of hexagonal (Sn,Cu,Ni)Ti_3_ increased from 13.8 wt % to 21 wt % after 30 min of milling. This process is possible if—due to Cu(Ni,Cu)Ti_2_ and CuNiTi_2_ decomposition—some Cu and Ni are transferred to (Sn,Cu,Ni)Ti_3_, apart from amorphization. After 90 min of milling the weight percent of this phase is the smallest (6 wt %), but its amount increases up to 29 wt % owing to further milling. The crystallite size of the (Sn,Cu,Ni)Ti_3_ phase also decreased exponentially, from 14.9–26.2 nm to ~1.2 nm (on the edge of amorphous phases field) during the full milling time scale (Figure 8a). The unit cell dimensions of this phase changed continuously during the milling process, which indicated that the composition changed as well (Table 1); however, no trend was established. The content of Sn in the bulk master alloy was 5 at.%, so the Sn content of the (Sn,Cu,Ni)Ti_3_ phase certainly did not correspond to the initial composition. More Cu and Ni atoms were introduced in this phase during milling, which was also indicated by the unit cell value variations (Table 1).

The amount of (Cu,Sn)Ti_3_, which was one phase of the eutectic, increased from 5.7 wt % to 22.4 wt % up to 60 min of milling time. This increase could only be explained if Ni and Ti, together with Cu, were also replacing Sn. Its weight fraction was almost halved after 120 min of milling, then increased to 19.7 wt %; these changes were observed inversely for (Sn,Cu,Ni)Ti_3_. The unit cell dimensions of this Cu(Sn)Ti_3_ phase changed continuously during the milling process, indicating that the composition changed too, although without an established trend. However, we assume that the evolution of (Sn,Cu,Ni)Ti_3_ and (Cu,Sn)Ti_3_ was interdependent of one another based on the quantitative changes within 30 and 120 min milling span.

Modeling amorphous components, or “short range ordered phases”, with the peak fitted to halo (hump) method was useful and allowed us to extract information on the composition of amorphous phases and size range of domains with different compositions. Observing the amorphous halo, the maximum position of the halo reflected the most frequent distances between atoms, so the peak corresponding to smaller angles corresponded with the amorphous phase with the largest distances between atoms [29]. Sn with a 0.1620 nm atomic radius and Ti with a 0.1462 nm atomic radius were much larger in this system compared to Cu or Ni; thus, amorphous phase detected by us was enriched with Sn and Ti. As shown by the first hump maximum evolution (Table 2), the distances between atoms decreased linearly during milling (Table 2, Figure 8a), which meant that the chemical composition of “short range ordered phases” changed, and the Ti and Sn content of amorphous phase was diluted by Cu and Ni. After 60 min of milling, the appearance of the second peak in amorphous halo indicated the formation of a secondary short-range order in the amorphous phase (Table 2). In this system, the X-ray scattering amplitudes of Ti and Cu were nearly equal, namely the appearance of inhomogeneity areas was much more pronounced [30]. This secondary short-range order phase was probably enriched in Cu and/or Ni, and its appearance coincided with the quantity reduction of (Sn,Cu,Ni)Ti_3_. The evolution of the two halo maximums was inversely related, which—in terms of atomic separation and crystallization—indicated Sn with Ti and Cu with Ni extraction from the amorphous phases, leading to the crystallization of (Sn,Cu,Ni)Ti_3_. The amorphous–nanocrystalline transition was a reversible process, as could be seen from the XRD results. Cyclic phase transitions could be observed, i.e., amorphization of a nanocrystalline alloy phase and crystallization of an amorphous alloy phase due to the applied high milling energy. Both short range ordered domain sizes decrease during the milling process, but sizes below 0.6 nm (roughly 5 neighboring atoms) were restricted in the fitting to avoid unrealistic approaches.

The α-Fe content was considered as contamination material of the milling process coming from the milling media, and it increased with the milling time up to 120 min. Medium-sized metallic atoms, such as Fe, have been widely used as alloying elements in various alloy systems. The maximal content evolved in our experiments was 4.2 wt % based on XRD measurement. However, the quantity of this element decreased suddenly after 90 min of milling, indicating that Fe atoms were dissolved in both crystalline and amorphous phases. The decrease in α-Fe phase content was a result of dissolving. The XRD method could not detect the dissolved Fe contamination. However, XEDS analysis confirmed that Fe content increased with milling time from 2.1 to 6.2 wt %. The dissolved Fe could have induced recrystallization processes of the amorphous phase [31]. The high Fe percentage indicates the need for studying the use of different milling media. Ni and Cr contamination also presented in the powder—Cr concentration was found around 0.8 wt % after 180 min milling time, while difference in Ni concentration was below measurement error.

Figure 8b illustrates the DSC scans for the amorphous–nanocrystalline Ti_50_Cu_25_Ni_20_Sn_5_ alloy samples after 180 min of milling. It should be mentioned that the crystallization peak observed for amorphous materials obtained by high-energy MM was usually wider and less intense than the corresponding one for the cast bulk metallic glasses or MA [32]. Two overlapping exothermic peaks could be observed, indicating multistage crystallization events. The DSC trace of partly amorphous alloy after 180 min of milling was slightly different from the DSC trace of melt-spin [20] or mechanical alloying [14] sample with the same nominal composition. Glass transition temperature (Tg) was not observed exactly in our case, indicating the difference between MM amorphous phase and metallic glass-type amorphous of MA. The onset temperature of crystallization was 450 °C, which was the lowest temperature value compared to the T_x_ of MA sample (474 °C) and melt-spin sample (492 °C).

## 4. Conclusions

Based on the experiments performed, the following conclusions can be reached:

1. Mechanical milling of Ti_50_Cu_25_Ni_20_Sn_5_ alloy by high-energy planetary ball milling up to 180 min led to the formation of nanocrystalline and amorphous composite. Amorphous fraction increased during milling due to intense shearing and plastic deformation induced by mechanical effect of balls.
2. After a short milling time (120 min), an amorphous structure with nanocrystalline phases of sizes in the 1.1–13.1 nm range was produced.
3. The maximal amorphous fraction (36 wt %) was obtained after 120 min of milling. Two short range ordered phases could be distinguished based on X-ray diffraction, indicating atomic segregation in the transforming solid phases.
4. Cyclic phase transformation took place during the milling process; Amorphization of a nanocrystalline alloy phase and recrystallization of an amorphous alloy phase occurred due to the high milling energy applied.
5. The crystallite size continuously decreased; the interval of crystallite size was between 1 and 10 nm after 180 min of milling.
6. Cubic Cu(Ni,Cu)Ti_2_ structure was transformed into the orthorhombic structure owing to the shear/stress, dislocations, and Cu substitution during the milling process.
7. Fe contamination from milling media increased with the milling time up to 90 min of milling, but longer milling time alloyed Fe into the milled material.

## Figures and Tables

**Table 1:** Features of crystallites based on the XRD of milled powder structure in the case of Ti_50_Cu_25_Ni_20_Sn_5_ alloy.

Phase	Milling Time, min	0	30	60	90	120	150	180
cubic **CuNiTi_2_** space group Pm-3m	a, nm	0.3050	0.3054	0.3089	0.3082	0.3100	0.3074	0.3069
	crystallite size, nm	16.2–25.5 ± 2.8	9.6–6.1 ± 1.3	3.1–4.8 ± 0.6	1.7–2.6 ± 0.5	1.5–2.3 ± 0.7	2.6–4.0 ± 0.3	2.7–4.3 ± 0.4
	wt % Rietveld	53.0	13.0	6.6	4.1	1.6	1.0	0.8
	cell volume, nm^3^	28.49	28.49	28.62	29.28	29.79	29.05	28.92
	R Bragg	3.60	2.26	2.31	2.82	74.63	29.58	8.95
orthorhombic **Cu(Ni,Cu)Ti_2_** space group Pmmb	a, nm	0.2990	0.2950	0.2950	0.2950	0.2907	0.2850	0.2850
	b, nm	0.4330	0.4300	0.4300	0.4300	0.4300	0.4300	0.4300
	c, nm	0.4460	0.4450	0.4450	0.4450	0.4450	0.4450	0.4450
	crystallite size, nm	10.5–16.4 ± 1.6	3.8–6.0 ± 0.6	2.0–3.1 ± 0.4	1.0–1.6 ± 0.4	1.1–1.8 ± 0.3	~1.3 ± 0.2	~1.0
	wt % Rietveld	13.3	33.4	24.8	31.3	34.5	35.0	25.7
	cell volume, nm^3^	57.76	56.45	56.45	56.45	55.61	54.53	54.53
	R Bragg	4.70	3.02	3.83	4.14	67.07	27.87	8.28
hexagonal **α-Ti(Cu)** space group P63/mmc	a, nm	0.2970	0.2967	0.2967	0.2967	0.2967	0.2967	-
	c, nm	0.4700	0.4700	0.4700	0.4700	0.4700	0.4700	-
	crystallite size, nm	6.4–10 ± 0.8	6.4–10 ± 1.5	6.2–9.7 ± 1.8	8.4–13.1 ± 5.8	8.8–13.1 ± 8.5	9.6–15.1 ± 11.8	-
	wt % Rietveld	13.7	11	7.5	3.2	1.6	0.8	0
	cell volume, nm^3^	35.83	35.83	35.83	35.83	35.83	35.83	-
	R Bragg	6.71	4.80	4.12	3.85	65.6	27.55	-
hexagonal **(Sn,Cu,Ni)Ti_3_** space group P63/mmc	a, nm	0.5894	0.5890	0.5890	0.5923	0.6303	0.6157	0.5834
	c, nm	0.4759	0.4765	0.4765	0.4764	0.4741	0.4730	0.5080
	crystallite size, nm	14.9–26.2 ± 3.8	7.7–13.7 ± 0.7	7.1–11.1 ± 1.3	6.7–10.5 ± 1.9	2.3–3.6 ± 0.8	1.4–2.2 ± 0.5	1.2 ± 0.2
	wt % Rietveld	14.2	20.9	9.8	6.0	10.5	17.3	29.1
	cell volume, nm^3^	143.18	143.16	143.16	144.72	163.11	155.26	149.71
	R Bragg	8.52	6.78	5.29	5.22	49.61	35.61	7.95
tetragonal **(Cu,Sn)Ti_3_** space group P4/mmm	a, nm	0.4150	0.4250	0.4219	0.4250	0.4250	0.4222	0.4250
	c, nm	0.3580	0.3500	0.3552	0.3535	0.3582	0.3600	0.3510
	crystallite size, nm	6.4–10 ± 1.5	3.2–5 ± 0.7	2.5–3.9 ± 0.5	2.4–3.8 ± 0.9	1.9–3 ± 0.7	1.8–2.8 ± 0.4	1.3–2.1 ± 0.3
	wt % Rietveld	5.8	15.0	22.0	17.2	13.7	35.0	25.7
	cell volume, nm^3^	61.64	63.22	63.22	63.85	64.70	64.18	63.41
	R Bragg	8.10	4.36	4.22	4.03	65.5	27.9	8.86
cubic **α-Iron** space group Im-3m	a, nm	-	0.2872	0.2873	0.2873	0.2873	0.2876	0.2883
	crystallite size, nm	-	9.8–15.4 ± 2.1	9.5–15.0 ± 2.5	7.4–11.6 ± 2.2	9.8–15.4 ± 3.3	6.4–10.0 ± 7.7	6.4–10.0 ± 4.9
	wt % Rietveld	-	2	3.8	4.2	2.3	0.7	0.7
	cell volume, nm^3^	-	23.70	23.72	23.71	23.71	23.78	23.96
	R Bragg	-	3.00	8.81	3.31	72.7	22.65	10.05

**Table 2:** Features of amorphous structure based on the XRD in the case of Ti_50_Cu_25_Ni_20_Sn_5_ alloy.

Milling Time, min	Amorphous Fraction, wt %	Amorphous Halo					
		First Peak			Second Peak		
		Position, nm	Size, nm	Area cps*2Th	Position, nm	Size, nm	Area cps*2Th
0	3	0.1891	1.0	2.2	-	-	-
30	5	0.2006	1.0	2.2	-	-	-
60	25	0.1938	0.8	10.4	0.1309	0.9	30.2
90	34	0.1841	0.6	17.7	0.1318	0.6	60.6
120	36	0.1781	0.6	32.1	0.1336	0.6	99.1
150	28	0.1646	0.6	44.4	0.1330	0.6	117.8
180	24	0.1608	0.7	36.1	0.1321	0.6	109.8
